# Targeting of tubulin polymerization and induction of mitotic blockage by Methyl 2-(5-fluoro-2-hydroxyphenyl)-1*H*-benzo[*d*]imidazole-5-carboxylate (MBIC) in human cervical cancer HeLa cell

**DOI:** 10.1186/s13046-016-0332-0

**Published:** 2016-03-31

**Authors:** Mohadeseh Hasanpourghadi, Chandrabose Karthikeyan, Ashok Kumar Pandurangan, Chung Yeng Looi, Piyush Trivedi, Kinue Kobayashi, Kozo Tanaka, Won Fen Wong, Mohd Rais Mustafa

**Affiliations:** Department of Pharmacology, Faculty of Medicine, University of Malaya, Kuala Lumpur, 50603 Malaysia; School of Pharmaceutical Sciences, Rajiv Gandhi Proudyogiki Vishwavidyalaya, Bhopal, 462033 India; Department of Molecular Oncology, Institute of Development, Aging and Cancer, Tohoku University, 980-8575 Sendai, Japan; Department of Medical Microbiology, Faculty of Medicine, University of Malaya, Kuala Lumpur, 50603 Malaysia

**Keywords:** Cervical cancer, Microtubule dynamics, Mitotic arrest, Mitochondrial-dependent apoptosis

## Abstract

**Background:**

Microtubule Targeting Agents (MTAs) including paclitaxel, colchicine and vinca alkaloids are widely used in the treatment of various cancers. As with most chemotherapeutic agents, adverse effects and drug resistance are commonly associated with the clinical use of these agents. Methyl 2-(5-fluoro-2-hydroxyphenyl)-1*H*- benzo[*d*]imidazole-5-carboxylate (MBIC), a benzimidazole derivative displays greater toxicity against various cancer compared to normal human cell lines. The present study, focused on the cytotoxic effects of MBIC against HeLa cervical cancer cells and possible actions on the microtubule assembly.

**Methods:**

Apoptosis detection and cell-cycle assays were performed to determine the type of cell death and the phase of cell cycle arrest in HeLa cells. Tubulin polymerization assay and live-cell imaging were performed to visualize effects on the microtubule assembly in the presence of MBIC. Mitotic kinases and mitochondrial-dependent apoptotic proteins were evaluated by Western blot analysis. In addition, the synergistic effect of MBIC with low doses of selected chemotherapeutic actions were examined against the cancer cells.

**Results:**

Results from the present study showed that following treatment with MBIC, the HeLa cells went into mitotic arrest comprising of multi-nucleation and unsegregated chromosomes with a prolonged G_2_-M phase. In addition, the HeLa cells showed signs of mitochondrial-dependant apoptotic features such as the release of cytochrome c and activation of caspases. MBIC markedly interferes with tubulin polymerization. Western blotting results indicated that MBIC affects mitotic regulatory machinery by up-regulating BubR1, Cyclin B1, CDK1 and down-regulation of Aurora B. In addition, MBIC displayed synergistic effect when given in combination with colchicine, nocodazole, paclitaxel and doxorubicin.

**Conclusion:**

Taken together, our study demonstrated the distinctive microtubule destabilizing effects of MBIC against cervical cancer cells *in vitro*. Besides that, MBIC exhibited synergistic effects with low doses of selected anticancer drugs and thus, may potentially reduce the toxicity and drug resistance to these agents.

**Electronic supplementary material:**

The online version of this article (doi:10.1186/s13046-016-0332-0) contains supplementary material, which is available to authorized users.

## Background

Cervical cancer is the third most common cancer and the fourth cause of death in women worldwide. More than 85 % of deaths due to cervical cancer occur mostly in developing countries. Globally, South-central Asia is reported to have among the highest incidences of cervical cancer [1]. In Malaysia, cervical cancer is rated as second most common cancer among women [2]. Human papillomavirus (HPV) infection is the main cause of cervical cancer [3]. By now it is well documented that cervical cancer cannot develop in the absence of the persistent HPV DNA [4].

Chemotherapy is a category of cancer treatment using one or more chemotherapeutic antitumor drugs. A more efficacious chemotherapy is expected when it is given to patients concurrently with radiation because chemotherapy and radiotherapy synergistically suppress DNA repair after the DNA damage caused by radiation [5]. In this regard, Cisplatin is a chemotherapeutic drug used to treat cervical cancer. Cisplatin-based chemotherapy with radiotherapy significantly improves survival for high-risk, early-stage cervical cancer patients. However, cisplatin displays acute and late toxicity which limits its therapeutic effectiveness. At the same time, many patients develop drug resistance to cisplatin after prolonged treatment [6]. Therefore, there is a need for a new anticancer agent with better efficacy and less toxicity.

Microtubule-targeting agents (MTAs) or microtubule inhibitors such as paclitaxel, docetaxel, vinblastine, estramustine, epothilones, colchicine, and nocodazole are commonly used in cancer chemotherapy [7]. Microtubules are the fundamental element of mitotic spindles and they are vital for numerous functions such as intracellular trafficking, maintenance of cellular architecture and cell movement and migration [8]. MTAs arrest cell cycle progression in mitosis by perturbing the microtubule dynamics and function [9]. MTAs interfere with microtubule dynamics and consequently, the formation of the spindle is disrupted and mitotic kinases function is restricted [10]. As a result, chromosomes are not able to attach to microtubule fibers precisely. In pursuance of protecting genome integrity and to reduce the occurrence of aneuploidy, the spindle assembly checkpoint delays metaphase-anaphase transition and causes mitotic arrest [11]. Following prolonged mitotic arrest, cancer cells either die in suspended mitosis through mitotic catastrophe [12], or exit mitosis without dividing (a process is known as mitotic slippage) to form multi-nucleated cells [13]. The effectiveness of MTAs has been hampered by the appearance of severe adverse effects particularly hematological and neurological toxicities and development of drug resistance. Development of new MTAs with decreased side effects and overcoming drug resistance may provide more effective therapeutic options for cancer patients [14].

Benzimidazoles are nitrogen heterocycles that contained a phenyl ring fused to an imidazole ring [15]. Benzimidazole derivatives are well documented as anticancer agents [16]. It was known that benzimidazole derivatives are potent inhibitors of tubulin polymerization [17]. Karthikeyan et al. [18] synthesized a series of 2-phenyl benzimidazole derivatives and showed that methyl 2-(5-fluoro-2-hydroxyphenyl)-1*H*-benzo[*d*]imidazole-5-carboxylate (MBIC) possessed potent cytotoxic effect in two breast cancer cell lines compared to cisplatin. In this study, we investigated the cytotoxic effect and the underlying molecular mechanistic action of the newly discovered antitumor agent, MBIC in HeLa human cervical adenoma cancer cells *in vitro*.

## Methods

### Reagents and chemicals

Colchicine (Cat#C9754), Nocodazole (Cat#M1404) and Paclitaxel (Cat#T1912) were purchased from Sigma-Aldrich (St. Louis, MO, USA). Doxorubicin HCI (Doxorubicin, Adriamycin®), (Cas#25316-40-9) was obtained from Pfizer, Inc (New York City, New York, USA). Stock solutions of selected tested compound (MBIC) were maintained in dimethyl sulfoxide (DMSO), protected from light and stored at −20 °C for experimental purposes.

### Test material

**Fig. 1:**
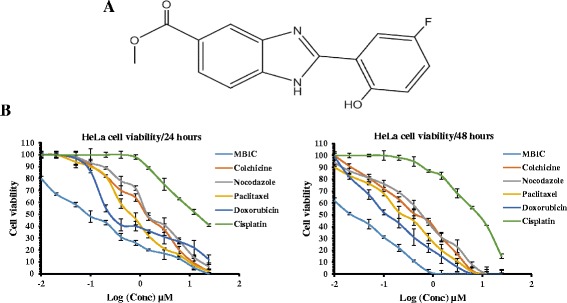
**a** Chemical Structure: Chemical structure of Methyl 2-(5-fluoro-2-hydroxyphenyl)-1H- benzo[d]imidazole-5-carboxylate (MBIC). **b** Inhibitory effect of MBIC against HeLa cell proliferation: Cell viability graph was generated for comparison of total relative cell viability (%) after MBIC and indicated conventional drugs treatment. Experiment was done in two time points (24 and 48 h) against HeLa cells. All results were expressed as total percentage of viable cells with mean ± SD of three independent experiments. (*P* < 0.05)

The synthesis of Methyl 2-(5-fluoro-2-hydroxyphenyl)-1H-benzo[d]imidazole-5-carboxylate (2e) has been described previously [18] and is also mentioned below. MBIC was kindly supplied by Professor Dr. Piyush Trivedi, School of Pharmaceutical Sciences, Rajiv Gandhi Technical University, India. The chemical structure of MBIC is shown in Fig. 1a.

### Procedure for the synthesis of Methyl 2-(5-fluoro-2-hydroxyphenyl)-1H-benzo[d]imidazole-5-carboxylate (MBIC)

To a solution of the 5-fluoro-2-hydroxybenzaldehyde (2 mmol) in 6.5 ml of N,N-dimethyl acetamide was added 3,4 methyl 3,4-diaminobenzoate (2 mmol) and Na_2_S_2_O_5_ (2.4 mmol). The mixture was heated to 100 °C for 8 h until TLC confirms the completion of the reaction. The reaction mixture was then cooled, diluted with ethyl acetate (5 × 25 ml), dried (MgSO_4_), and concentrated in vacuo. The solid obtained was collected on a sintered-glass filter and washed with dichloromethane (3x) to provide the desired compound.

Seventy two percent yield; M.P. 239 °C; IR (KBr) ν (cm^−1^): 3333 (–NH), 3057 (Ar–C–H), 1722 (–C = O), 1255 (–C–N), 1213 (–CO–O) 1535 (Ar C–C); ^1^H NMR (400 MHz, DMSO-d_6_): δ (ppm) 13.12 (br s, 1H, NH), 8.22 (s, 1H, Ar H), 7.82–7.88 (m, 2H, Ar H), 7.67(s, 1H, Ar H), 6.96–7.12 (m, 2H, Ar H), 3.85 (s, 3H, CH_3_); LC–MS analysis (M - H)^−^: 285.0 (calculated 286.08); Elemental Analysis: Calcd. (Found) (%) for C_15_H_11_FN_2_O_3_: C 62.94 (62.90), H 3.87 (3.89), N 9.79 (9.80) [18].

### Cell culture

The human cervical cancer cell-line, HeLa (CCL-2.2; ATCC, Manassas, VA), HCT-116 (CCL-247; ATCC, Manassas, VA), A549 (CCL-185; ATCC, Manassas, VA), HepG-2 (HB-8065; ATCC, Manassas, VA) and WRL-68 (CL-48; ATCC, Manassas, VA), were obtained from American Type Culture Collection (ATCC). HeLa, A549, HepG2 and WRL68 cells were maintained in Dulbecco’s Modified Eagle Medium (DMEM, Life Technologies, Inc, Rockville, MD). HCT-116 was obtained in McCoy’s 5a Medium Modified (Life Technologies, Inc, Rockville, MD) supplemented with 10 % fetal bovine serum and 1 % penicillin/streptomycin. Cells were incubated at 37 °C in a humidified cell culture 5 % CO_2_ incubator. Cells were passaged until reaching 70-80-% confluency for experimental purposes.

### Cytotoxicity assay

Cytotoxicity of MBIC was estimated by MTT assay. Briefly, 1 × 10^4^ cells/well were seeded in a 96-well plate and incubated at 37 °C in 5 % CO_2_. After 24 h, cells were treated with different concentrations (0.02, 0.04, 0.1, 0.2, 0.4, 0.8, 1.5, 3, 6, 12, 25, 50 μM) of MBIC. 0.01 % DMSO was used as vehicle control. Cells were incubated for 24 and 48 h at 37 °C. Subsequently, 50 μl/well of 3-(4,5-dimethylthiazol-2-yl)-2,5-diphenyltetrazolium bromide (MTT, 2 mg/ml) was added and incubated for 2 h. After the incubation, the media was discarded and 100 μl DMSO was replaced into each well to dissolve the formazan crystal. The colorimetric assay was quantified at 570 nm wavelength using Chameleon V microplate reader (Hidex, Turku, Finland). The anti-proliferation activity of MBIC was expressed as an IC_50_ value. The percentage of cell viability was calculated as described previously [19].

### Apoptosis analysis

Apoptosis was detected using BD Pharmingen Annexin V-FITC Apoptosis Detection Kit as per manufacturer’s protocol. Briefly, cells were seeded in 25 cm^2^ culture flasks (10 × 10^5^ cells/flask) and were treated in 3 different MBIC concentrations for 24 and 48 h. Later, treated cells were harvested and washed with 1 ml of 1X Phosphate Buffered-Saline (PBS) before adding 200 μl 1X annexin-binding buffer. 10 μl of annexin V-FITC and 10 μl of PI were added to each 200 μl of cell suspension. Cells were then incubated in total darkness for 15 min at room temperature. 500 μl of 1X annexin-binding buffer was added just before the flow cytometry analysis.

### Cell cycle analysis

Cell cycle analysis was performed using propidium iodide (PI). Briefly, cells were seeded in a 25 cm^2^ flask, then 60 % confluent cells were treated with MBIC for 24 and 48 h. Following incubation, the cells were harvested and spun down for 5 min at 2000 rpm. The supernatant was removed and cells were then fixed in 1 ml of ice-cold 70 % ethanol overnight at -80 °C. Fixed cells were washed with 1 ml 1X PBS and stained in 500 μl of PI containing 5 μg/ml DNase-free RNase for 30 min at room temperature in total darkness. DNA content of the cell was analyzed by flow cytometry. The percentage of G_0_-G_1_, S and G_2_-M cells were then calculated using Fluorescence-activated cell sorting (FACS) software (BD Biosciences).

### Live-cell imaging

HeLa cells expressing EGFP-α-tubulin, EGFP-CENP-A and histone H2B-mCherry were grown in glass chambers (Thermo Scientific, USA). Thirty minutes before imaging, the medium was changed to pre-warmed Leibovitz’s L-15 medium (Life Technologies) supplemented with 20 % fetal bovine serum and 20 mM HEPES, pH 7.0. Recordings were made in a temperature-controlled incubator at 37 °C. Images were collected with an Olympus IX-71 inverted microscope (Olympus) controlled by DeltaVision softWoRx (Applied Precision) using a 20x 0.75 NA UPlan SApochromat objective lens (Olympus). Z-series of six sections in 3 μm increments were captured every 15 min. Image stacks were projected.

### Tubulin polymerization assay

To investigate the effect of MBIC on tubulin polymerization, a fluorescence-based tubulin polymerization assay kit (Cytoskeleton-Cat. # BK011P) was used according to the manufacturer’s protocol. Briefly, 200 μl of pure tubulin protein was re-suspended in 420 μl of ice cold Tubulin Polymerization Buffer (TPB) to give a final concentration of 3 mg/ml tubulin, supplemented with 80 mM PIPES, 2 mM MgCl_2_, 0.5 mM EGTA, 1 mM GTP, 20 % (*v*/*v*) glycerol. 100 μl of tubulin reaction mixture were added to each well in a 96-well plate containing the selected concentration of MBIC (10 μM). Paclitaxel, colchicine, nocodazole were applied separately as positive controls (10 μM). Samples were mixed well and the tubulin assembly was monitored by an increase in fluorescence emission at 340 nm in kinetic mode for 120 min at 37 °C using a plate reader (Infinite® 200 PRO—Tecan plate reader, USA).

### Western blot analysis

Western blot analysis was used to investigate protein expression levels. It was adopted by Suresh Kumar et al. [20]. Briefly, 1 × 10^5^ cells/flask was seeded and treated with MBIC in a dose-dependent manner for 24 h. Cells treated with colchicine and nocodazole served as positive controls. Cells were collected and lysed in RIPA buffer supplemented with a 10 μl protease inhibitor cocktail, sodium orthovanadate, and PMSF (Santa Cruz, USA). The lysate was stored at −80 °C until further use. A 40 μg of sample protein was resolved on 10 % SDS-PAGE and transferred to a polyvinylidene difluoride (PVDF) membrane (Merck Millipore, USA). The membrane was blocked in 5 % BSA for 1 h at room temperature following an overnight incubation at 4 °C with the following primary antibodies: Anti-Cyclin B1, anti-CDK1, anti-BubR1, anti-Aurora B, anti-cleaved PARP, anti-cleaved caspase-3/7/9, anti-Bcl-2 and anti-Bax (1:1000) (Cell Signaling Technology (CST), USA), mouse anti β-actin (1:40000) (Sigma-Aldrich, USA) antibodies.

Membranes were washed with 1X TBS-T prior to incubating with HRP-conjugated goat anti-mouse or anti-rabbit secondary antibodies for 2 h at room temperature. Membranes were washed 3 times with 1X TBS-T to remove excess antibodies before the proteins-antibody complex was detected with Amersham ECL prime western blotting detection reagent (GE Healthcare, USA). Western blot images were quantified and processed by ImageJ software (NIH, USA).

### Multiparameter cytotoxicity assay

The critical apoptotic events in cervical cancer including cell permeability, mitochondrial membrane potential (MMP), cytochrome c release and total nuclear intensity after treatment with MBIC were determined using the Cellomics Multiparameter Cytotoxicity 3 Kit. Briefly, 1 × 10 ^4^ cells/well were seeded in a 96-well plate and incubated for 24 h prior to treatment with MBIC at various concentrations. At 24 h post treatment, cells were stained with cell permeability and MMP dye and further incubated for 1 h. Stained cells were fixed and permeabilized with 4 % formaldehyde and 0.1 % Triton X-100 in PBS, respectively. Cells were washed twice with 1X PBS prior to blocking with 3 % bovine serum albumin.

Cells were rinsed twice with Wash Buffer I (1X PBS) and incubated with 50 μl/well of Cytochrome c primary antibody. After 60 min, the plate was washed with 1X Wash Buffer and subsequently, 50 μl of goat anti-mouse secondary antibodies conjugated with DyLight 649 were added into each well. Cells were rinsed twice with Wash Buffer I (1X PBS). Hoechst 33342 was added into the staining solution to stain the nucleus. Stained cells in the 96-well plates were analyzed using ArrayScan High Content Screening (HCS) system (Thermo Scientific, USA).

### Drug combination assay

In order to discover the synergistic effects of MBIC with conventional drugs briefly, cells were seeded in 1 × 10^4^ cells/well and were incubated at 37 °C overnight. Thereafter, cells were treated with the selected conventional drugs—paclitaxel, colchicine, nocodazole and doxorubicin separately in various concentrations. Also, the MBIC, combined with each of the selected conventional drugs in a 1:1 ratio, were diluted in concentration from 50 μM to 0.1 μM. Cell viability assay (MTT assay) was performed 24 h post-treatment and absorbance were measured in a microplate reader to find out the IC_50_ for each conventional drug alone and in combination with MBIC on HeLa cells. For this analysis, we used the commercial software package CampuSyn (Biosoft, Cambridge, United Kingdom; Ref.9). After entering doses and effects of MBIC and other drugs either separately or combined, a normalized combination index (CI) and dose reduction index (DRI) with values of 50 % to 97 % inhibition was obtained. Furthermore, the software produced an automated isobologram and fraction affected-combination index (Fa-CI) plot.

### Statistical analysis

Statistical analysis was processed according to conventional procedures using the Statistical Program for Social Sciences (SPSS) software for Windows, Version 12.0 (Post-hoc, Turkey’s test). A *P* value <0.05 was considered statistically significant.

## Results

### MBIC is cytotoxic against different human cancer cell lines

MTT assays were performed to evaluate the inhibitory activity of MBIC on HCT-116 (colorectal), A549 (lung), HepG-2 (hepatocellular), HeLa (cervical) cancer cell lines compared to human embryonic normal liver cell line (WRL-68). We found that the half maximal inhibitory concentration (IC_50_) of MBIC on human cancer cell lines was <5 μM, comparable to several conventional drugs such as colchicine, nocodazole, paclitaxel, doxorubicin and cisplatin (Table 1). Among the cancer cell-lines, MBIC demonstrated the highest cytotoxicity against HeLa cells, for 24 and 48-h treatment time point. In addition, MBIC showed better selectivity in HeLa cells (>30 fold) compared to other conventional drugs (Table 1).

**Table 1:** Inhibitory effect of MBIC against human cancerous and non-cancer cell-lines

Cell line	MBIC		Colchicine		Nocodazole		Paclitaxel		Doxorubicin		Cisplatin	
	IC_50_	SI	IC_50_	SI	IC_50_	SI	IC_50_	SI	IC_50_	SI	IC_50_	SI
IC_50_ (μM)/24 hours												
HeLa	0.21 ± 0.02	48.04	3.01 ± 0.03	2.66	3.08 ± 0.02	3.97	1.02 ± 0.01	6.57	0.46 ± 0.04	7.73	15.07 ± 0.03	1.23
HCT-116	2.72 ± 0.4	3.70	5.34 ± 0.2	1.50	3.81 ± 0.6	3.20	3.09 ± 0.5	2.17	0.91 ± 0.2	3.91	7.22 ± 0.5	2.57
A549	1.29 ± 0.07	7.82	7.78 ± 0.3	1.03	12.16 ± 0.2	1.00	2.83 ± 0.11	2.37	0.35 ± 0.07	10.17	34.04 ± 0.9	0.54
HepG-2	2.92 ± 0.1	3.45	8.36 ± 0.9	0.95	10.71 ± 0.5	1.14	2.81 ± 0.72	2.38	8.04 ± 0.8	0.44	10.31 ± 0.5	1.80
WRL-68	10.09 ± 1.2		8.02 ± 0.5		12.23 ± 0.9		6.71 ± 0.2		3.56 ± 0.9		18.6 ± 1.4	
IC_50_ (μM)/48 hours												
HeLa	0.19 ± 0.03	37.68	2.02 ± 0.02	2.04	2.22 ± 0.04	3.40	0.57 ± 0.03	5.98	0.29 ± 0.04	5.79	9.02 ± 0.02	1.17
HCT-116	2.01 ± 0.3	3.56	3.44 ± 0.2	1.20	1.81 ± 0.3	4.17	1.09 ± 0.4	3.12	0.47 ± 0.3	3.57	4.12 ± 0.6	2.56
A549	0.53 ± 0.1	13.50	3.21 ± 0.7	1.28	5.09 ± 0.5	1.48	1.23 ± 0.09	2.77	0.09 ± 0.01	18.66	19.51 ± 1.4	0.54
HepG-2	1.03 ± 0.09	6.95	4.08 ± 0.4	1.01	5.08 ± 0.3	1.48	1.03 ± 0.07	3.31	3.94 ± 0.07	0.42	5.93 ± 0.2	1.78
WRL-68	7.16 ± 0.9		4.14 ± 0.7		7.56 ± 0.3		3.41 ± 0.8		1.68 ± 0.09		10.56 ± 0.7	

Human cervical cancer (HeLa), colorectal (HCT-116), lung cancer (A549), hepatocellular (HepG-2) cell-lines and human embryonic normal liver cell line (WRL-68) were treated with DMSO as vehicle control, various concentration of MBIC and selected conventional drugs (colchicine, nocodazole, paclitaxel, doxorubicin and cisplatin) for 24 and 48 h. Cell viability was determined by MTT assay and by calculation of half maximal inhibitory concentration (IC_50_). Data were mean ± SD of three independent experiments. (*P* < 0.05). Selectivity Index (SI) is calculated by dividing IC_50_ of non-cancer cell-line (WRL-68) against IC_50_ of human cancer cell-lines

### MBIC induced apoptosis

**Fig. 2:**
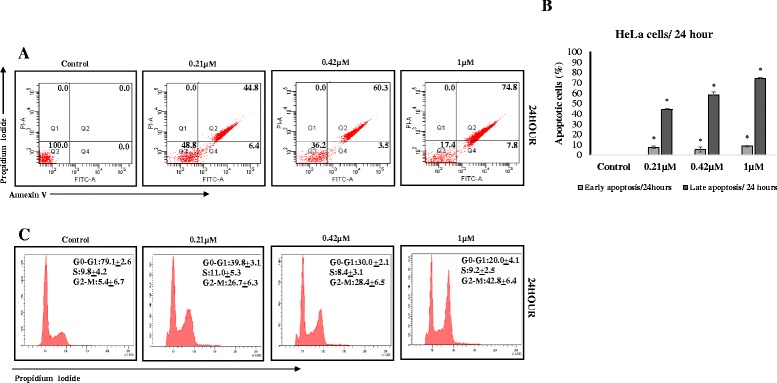
**a** MBIC induced apoptosis in HeLa cells: Flow cytometry analysis of HeLa cells treated with various concentration of MBIC for 24 h was carried out. Representative figures show population of viable cells in Q3 (annexin V- PI-), early apoptotic cells in Q4 (annexin V+ PI-), late apoptotic cells in Q2 (annexin V+ PI+) and necrotic cells in Q1 (annexin V- PI+). Representative figure shows apoptosis induction of MBIC (0.21, 0.42 and 1 μM) against HeLa cells 24 h after treatment. **b** shows early and late induced apoptosis in bar chart for HeLa cells 24 h after treatment. Data were mean ± SD of three independent experiments. All the treatment groups were compared with control. “*” indicates statistically significant at *P* < 0.05. **c** MBIC induced G_2_-M arrest in HeLa cells: HeLa cells were treated with indicated concentrations of MBIC (0.21, 0.42 and 1 μM) for 24 h. Cells were permeabilized by ethanol and stained with PI. Cell cycle progression has been assessed by flow cytometry. Representative figures of cell cycle distribution (G_0_-G_1_, S, and G_2_-M) show accumulation of MBIC-treated cells in G_2_-M phase. HeLa cells were arrested in G_2_-M phase 24 h after MBIC treatment

Since MBIC demonstrated higher cytotoxicity and selectivity in HeLa, subsequent assays were performed using this cancer cell-line. During early apoptosis, membrane phosphatidylserine (PS) translocate from the inner face of the cell membrane to the cell surface. Annexin V can bind to exposed PS with high affinity, whereas PI molecules intercalate inside the DNA double helix in cells with a compromised plasma membrane. Therefore, cells stained strongly with Annexin V signifies early apoptosis and PI-stained cells indicate late apoptosis or necrosis [21]. To examine whether MBIC-treated HeLa cells undergo apoptosis or necrosis, MBIC treated cells were stained with annexin V and PI. As shown in Fig. 2a, MBIC exposure at different concentrations (0.21, 0.42 and 1 μM) resulted in a higher population of late apoptotic cells (44.8 ± 2.3 % to 74.8 ± 4.2 %) compared to control (0.0 ± 0.0 %). Our results indicated that MBIC-induced dose-dependent apoptosis in HeLa cells as shown in the bar graphs (Fig. 2b).

### MBIC induced cell cycle arrest in G_2_-M phase

To investigate the cell cycle profile after MBIC treatment, we performed a cell cycle assay by staining HeLa cells with PI and analyzed the percentages of G_0_-G_1_, S and G_2_-M cell population using flow cytometry. HeLa cells were treated with MBIC for 24 h at the concentrations of 0.21, 0.42 and 1 μM of MBIC showed higher G_2_-M population (26.7 ± 6.3 % to 42.8 ± 6.4 %) compared to 5.4± 6.7 % in untreated cells (Fig. 2c).

### MBIC disrupts mitotic spindle

**Fig. 3:**
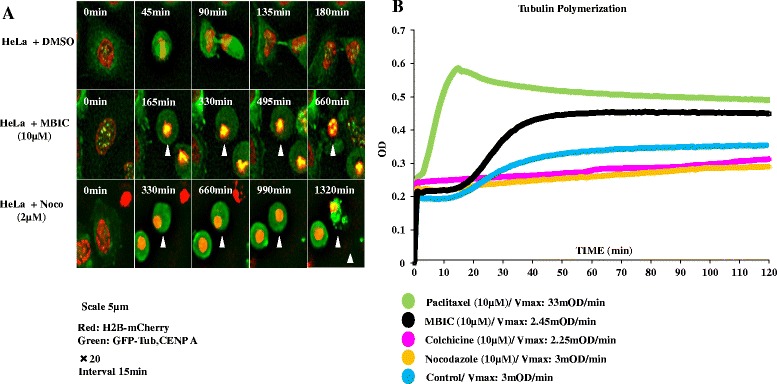
**a** MBIC disrupts mitotic spindle: HeLa cells expressing EGFP-α-tubulin, EGFP-CENP-A and histone H2B-mCherry were treated with DMSO (upper), MBIC (10 μM, middle), or nocodazole (2 μM, lower) and imaged at 15 min intervals. Time (min) is indicated in the upper left of each panel. For middle and lower panels, arrowheads mark the cell in the center at the first frame. Scale bar = 5 μm. **b** Effect of MBIC on tubulin polymerization: Tubulin polymerization assay was conducted using Tubulin polymerization assay kit (Cytoskeleton, Inc.). The plate was read using Infinite® 200 PRO—Tecan regulated on 96 well plate reader spectrophotometer. The plate was read at 340 nm in kinetic mode for two hours. Figure 3 shows curves of tubulin treated by paclitaxel (10 μM), nocodazole (10 μM), colchicine (10 μM), MBIC (10 μM) and untreated tubulin. Maximal velocity (Vmax) of each drug on tubulin polymerization was calculated

As cells were arrested in G_2_-M phase, we decided to examine MBIC’s action against microtubule dynamics and spindle formation in live-cell imaging. We observed HeLa cells stably expressing EGFP-α-tubulin, EGFP-CENP-A and histone H2B-mCherry (Fig. 3a). Control cells treated with DMSO formed bipolar spindle with aligned chromosomes (Fig. 3a, upper, 45 min) and segregated chromosomes properly without delay (Fig. 3a, upper, 90 min). In contrast, cells treated with MBIC did not form the spindle and stayed in mitosis for a long time before dying with pyknosis and cell shrinkage, i.e., characteristics of apoptotic cell death (Fig. 3a, middle), similar to cells treated with nocodazole (Fig. 3a, lower). The result indicated that MBIC disrupts spindle formation, consistent with its role as a MTA.

### MBIC inhibits microtubule polymerization

Next, we evaluated the effect MBIC on tubulin nucleation and polymerization. MBIC was applied into tubulin buffer (10 μM). Conjointly, we compared MBIC’s activity with several conventional MTA drug activities, such as paclitaxel, nocodazole and colchicine at 10 μM/well (Fig. 3b). Maximal velocity (Vmax) is a measurement showing how fast a drug can act on the substrate tubulin in a polymerization assay [22]. In an untreated sample, the Vmax is 12mOD/min. In a sample treated with MBIC, we found that MBIC interfered with tubulin nucleation phase (Vmax for 10 μM MBIC is 2.45mOD/min) comparable to the destabilizing activity of colchicine (Vmax:2.25mOD/min) and nocodazole (Vmax:3mOD/min). In contrast, paclitaxel (stabilize microtubules polymers) showed Vmax at 33mOD/min (Fig. 3b).

### Effect of MBIC on cell-cycle related proteins

**Fig. 4:**
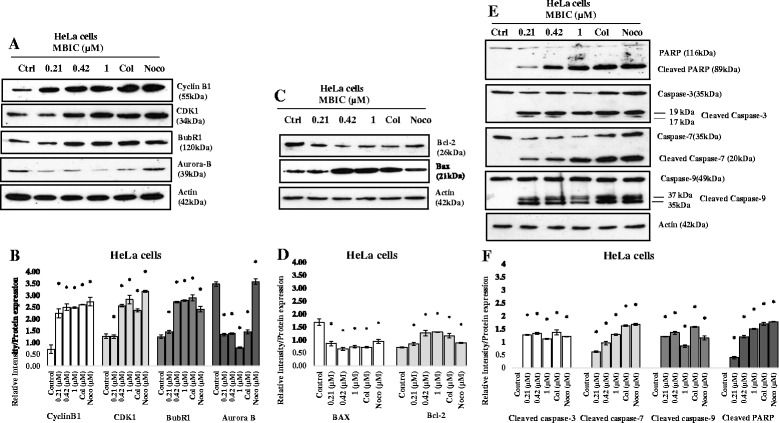
**a** MBIC induced mitotic arrest: Western blot analysis was carried out to evaluate mitotic regulators levels after MBIC treatment. HeLa cells were treated with (0.21, 0.42 and 1 μM of MBIC. Figure **a** shows the evaluation of Cyclin B1, CDK1, mitotic checkpoint protein (BubR1) and a chromosomal passenger complex member, Aurora B. Samples were treated with colchicine (Col) and nocodazole (Noco) (at their IC_50_ concentration) as a positive control. β-actin served as a loading control. **c**, **e** MBIC induced mitochondria-dependent apoptosis: In order to evaluate whether MBIC caused mitochondria-dependent apoptosis, we evaluated anti-apoptotic protein Bcl-2, pro-apoptotic protein Bax, cleaved caspase-3/7/9 and cleaved PARP levels by western blot analysis (Fig. 4 **c** & **e**). HeLa cells were treated with MBIC (0.21, 0.42 and 1 μM). Samples were treated with colchicine (Col) and nocodazole (Noco) (at their IC_50_ concentration) as a positive control. β-actin served as a loading control. **b**, **d** & **f** The relative intensity of each protein was normalized with β-actin. Data were mean ± SD of three independent experiments. All the treatment groups were compared with control. “*” indicates statistically significant at *P* < 0.05

Since cell cycle is governed by a group of proteins called cyclin-dependent kinases (CDKs) and mitotic kinases, we performed Western blot analysis to examine whether MBIC affects these targets. Cells were also treated with colchicine or nocodazole as positive controls. First, we decided to evaluate Cyclin B1 and CDK1 levels. Cyclin B1-CDK1 complex is known as a mitosis-promoting factor (MPF). Also, this complex is inactive in G_2_ phase and its activation begins exactly before nuclear envelope breakdown which leads on to set up the events in prophase [23]. As shown in Fig. 4a, we observed up-regulation of Cyclin B1 and CDK1, indicating the occurrence of mitotic arrest.

Next, we decided to evaluate the protein level of BubR1 and Aurora B. BubR1 is a spindle checkpoint protein which blocks metaphase-anaphase entry until all kinetochores are successfully attached to the spindle microtubules [24]. Aurora B is a member of the chromosome passenger complex (CPC). CPC activity is required to regulate the correct kinetochores to microtubules attachment to ensure proper mitotic checkpoint function [25]. Our results showed that BubR1 is up-regulated and Aurora B is down-regulated in treated HeLa cells (Fig. 4a), suggesting mitotic arrest after MBIC treatment.

### MBIC induced mitochondrial-dependant apoptosis

Since in apoptosis machinery mitochondrial-dependent proteins are also involved, we decided to examine the protein level of those enzymes associated with mitochondrial-dependant apoptosis. Western blot results showed up-regulation of Bax protein levels and down-regulation of Bcl-2 protein level (Fig. 4c). MBIC also increased the level of cleaved (activated) caspase-3/7/9 in treated HeLa cells, a hallmark of intrinsic apoptosis (Fig. 4e). Next, we decided to evaluate one of the most important caspase substrates, poly (ADP-ribose) polymerase (PARP) level. PARP is involved in the DNA repair process and the cleaved form of this protein prevents DNA repair after damage [26]. Our observation of an increased level of cleaved PARP, adds to the evidence that MBIC treatment initiates mitochondria-dependant apoptosis.

### Effect of MBIC on nuclear morphology, membrane permeabilization and mitochondria membrane potential (Δψm)

**Fig. 5:**
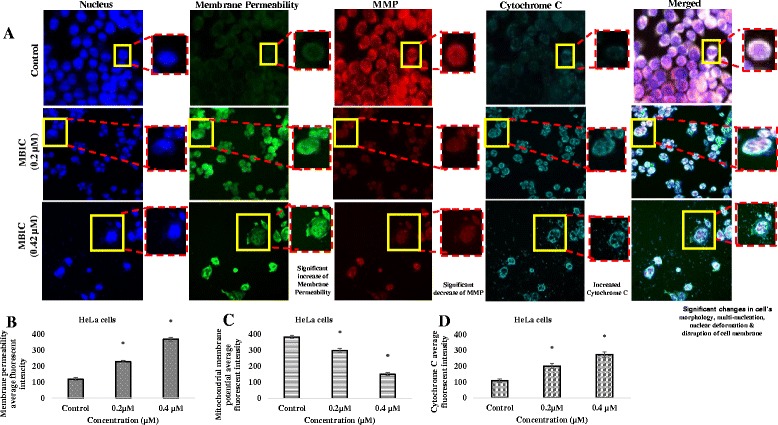
**a** Effect of MBIC on nuclear morphology, membrane permeabilization, MMP (Δψm) and cytochrome c release: HeLa cells were seeded in 96-well plate and treated with two different concentrations of MBIC (0.2 and 0.4 μM) for 24 h. Cells were fixed and stained according to manufacturer protocol. Images were acquired using Cellomics HCS array scan reader (objective 20 ×). Figure **a** displays changes in HeLa cells DNA content (*blue*), cell permeability (*green*), MMP (*red*) and cytochrome c (*cyan*). MBIC induced a considerable elevation in membrane permeability and cytochrome c release and caused an extensive reduction in mitochondrial membrane potential. **b, c** & **d** shows bar chart representing changes in membrane permeability, mitochondrial membrane potential, and cytochrome c in a dose-dependent manner. Data were mean ± SD of three independent experiments. All the treatment groups were compared with control. “*” indicates statistically significant at *P* < 0.05

**Fig. 6:**
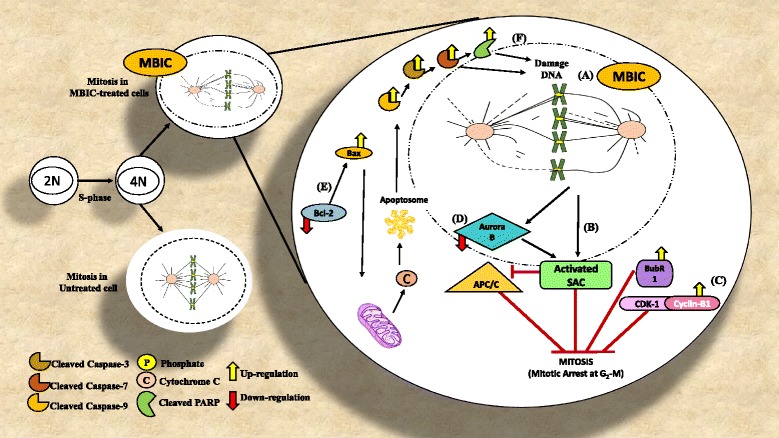
Proposed molecular mechanistic action of MBIC in HeLa cancer cell. **a** MBIC causes microtubule-kinetochore attachment error by interrupting microtubules polymerization. **b** Unattached kinetochores activate spindle assembly checkpoint (SAC). When SAC is activated, inhibits APC/C. BubR1 is a SAC member which inhibits metaphase to anaphase transition until all kinetochores are attached to microtubules correctly. Up-regulation of BubR1 is a sign of attachment error. **c** Cyclin B1 degradation is compulsory for exiting from mitosis. APC/C is responsible for Cyclin B1 degradation. Up-regulation of Cyclin B1 in our study shows that APC/C is not activated. **d** Down-regulation of Aurora B, one on chromosomal passenger complex (CPC) impairs SAC activity. **e** Down-regulation of Bcl-2 and up-regulation of Bax are signs of release of cytochrome c in cytosol and formation of apoptosome which activated caspase-9. Up-regulation of cleaved caspase-3/7/9 is a sign of mitochondrial-dependent apoptosis. **f** Up-regulation of cleaved PARP is a sign of DNA damage

To investigate further the sub-cellular changes induced by MBIC, we compared the nuclear morphology, membrane permeability, mitochondrial membrane potential (MMP, Δψm) and DNA content between treated and untreated cells. Compared to untreated cells, 24 h post-MBIC treatment (0.2 and 0.42 μM), HeLa cells showed a dose-dependent loss of cells, an increase of membrane permeability, exhaustion of MMP compared to untreated cells (Fig. 5a and b). On the other hand, the nucleus of untreated samples remained rounded and uniform in size. MMP and cytochrome c were stained consistently and co-localized in the cytosol, indicating no cytochrome c release in control cells. Fig. 6 is proposing the overall mechanistic action of MBIC in HeLa cell.

### MBIC displays a synergistic effect with selected conventional drugs

Combinations of drugs have been applied for treating different diseases such as cancer. Application of multiple drugs with synergistic effect may target multiple biomolecules functions, multiple molecular pathways, or multiple diseases simultaneously. Different modes of action among combined drugs, may also direct and magnify the effect against a single target or a single disease and thus, show more effective outcome [27]. In this study, we performed synergistic studies that were followed by Chou-Talalay method to evaluate a few combinations of conventional drugs with MBIC [28]. The Combination index (CI) quantitatively shows synergism if CI is smaller than 1 (CI < 1) and an additive effect if CI equals 1 (CI = 1). If CI is higher than 1(CI > 1), the CI shows antagonism. In this study, we combined MBIC with several conventional drugs such as paclitaxel, colchicine, nocodazole, and doxorubicin. As shown in Table 2, combined with each of our selected conventional drugs in a ratio of 1:1, HeLa cells treated with MBIC showed a synergistic effect (CI < 1). This synergistic effect ranged from 50 % inhibition to 97 %. Furthermore, software generated dose reduction index (DRI) of MBIC in combination with selected conventional drugs (Table 2). The DRI indicates the possible minimum dosage for each drug in the case of synergism [28]. For example in our study, MBIC in combination with colchicine had the highest DRI. Colchicine and MBIC’s DRI for 50 % inhibition were 165.4-fold and 9.9-fold, respectively. In the case of 97 % inhibition, the DRIs were 1883.3- and 44.1-fold, respectively. Additional file 1: Figure S1 displays isobologram and fractional affected-combination index (Fa-CI) plots to evaluate drug interaction in combination therapy.

**Table 2:** MBIC has synergistic effect in combination with selected conventional drugs

Drug combination	Combination ratio	(Dm)IC_50_ (μM)			CI values at inhibition of					DRI values at inhibition of				
		Drug 1	Drug 2	COMBINED	50 %	75 %	90 %	95 %	97 %	50 %	75 %	90 %	95 %	97 %
										(1)	(1)	(1)	(1)	(1)
										(2)	(2)	(2)	(2)	(2)
Colchicine + MBIC	1:1	3.01 ± 0.7	0.21 ± 0.02	0.076 ± 0.03	0.10	0.06	0.04	0.02	0.02	165.4	356.8	769.7	1298.3	1883.3
										9.9	15.8	25.4	35.1	44.1
Nocodazole + MBIC	1:1	3.08 ± 0.5	0.21 ± 0.02	0.087 ± 0.02	0.27	0.20	0.14	0.11	0.10	20.6	31.1	46.7	61.7	75.2
										4.3	5.8	7.9	9.7	11.2
Paclitaxel + MBIC	1:1	1.02 ± 0.6	0.21 ± 0.02	0.104 ± 0.06	0.35	0.28	0.23	0.20	0.18	15.3	18.4	22.1	25.0	27.3
										3.4	4.2	5.1	5.9	6.5
Doxorubicin + MBIC	1:1	0.46 ± 0.7	0.21 ± 0.02	0.092 ± 0.03	0.30	0.22	0.17	0.14	0.12	12.5	17.7	25.0	31.6	37.3
										4.4	5.7	7.5	9.1	10.3

Table 2 displays synergistic effects of MBIC in combination with paclitaxel, colchicine, nocodazole and doxorubicin against HeLa cells by using CampuSyn software (Biosoft, Cambridge, United Kingdom; Ref. 9). Combined drugs were in 1 to 1 ratio. After entering dose and effects of combined drugs individually and in combination, software generates Combination Index (CI) value at 50 to 97 % inhibitions of HeLa cells treated with combined drug (1) (conventional drugs) and drug (2) (MBIC). Additionally, table contains dose reduction index (DRI) for drug (1) and drug (2) which suggest how many fold of each drug in combination can be reduced while having same effect. Data were mean ± SD of two independent experiments. (*P* < 0.05)

## Discussion

Emergence of drug resistance and severe side effects limit the effectiveness of many anticancer drugs including microtubule targeting agents (MTAs). Among the many side effects of MTAs, those that interfere with the cell division of normal cells as well as cell division of cancer cells, are considered [29]. Therefore, discovery of new improved MTAs with less toxicity in normal cells are highly desirable in cancer chemotherapy [30]. Another drawback obstacle against MTAs effectiveness is the rise of drug resistance. One of the identified drug resistance is overexpression of multidrug resistance gene (MDR1) such as P-glycoprotein (P-gp) efflux pump which decreases intracellular drug levels. MDR1/P-gp-dependant docetaxel resistance in breast cancer cells has been reported [31]. Therefore discovery, design, and development of new MTAs, understanding their mechanisms of action and introducing new treatment strategies that decrease side effects and drug resistance, may afford improved therapeutic options for cancer patients [14].

Benzimidazole-derived scaffolds are receiving attention in cancer chemotherapy because of the presence of a heterocyclic imidazole ring that provides excellent possibilities for generating novel compounds as potential anticancer agents [32]. In the present study, we identified a new anti-cancer agent, MBIC that inhibits cancer cell proliferation with a low IC_50_ (IC_50_: 0.21 ± 0.02 μM) against HeLa cancer cells in comparison with selected standard drugs, but also has an advantage of a high IC_50_ (low cytotoxicity) against human normal liver cells (WRL-68) (IC_50_: 10.09 ± 1.2 μM). The present result suggests that MBIC interferes with tubulin dimers and disrupts tubulin assembly which is comparable with colchicine.

Agents that perturb microtubule assembly/disassembly represent an important therapeutic group in cancer chemotherapy [33]. These microtubule inhibitors, such as taxanes, stabilize microtubules polymerization and prevent cancer cell division [34]. In contrast, some other MTAs such as vinca alkaloids, colchicine, and nocodazole inhibit the assembly of tubulin dimers [22]. Previously, it was reported that some benzimidazole derivatives, such as oxibendazole and fenbendazole prevent tubulin polymerization and are colchicine competitors [35]. MBIC is a potential MTA with not only a greater toxicity against HeLa cancer cells in comparison with selected standard drugs but also has an advantage of a low toxicity against normal human liver cells (WRL-68). Further our results showed that MBIC interrupted tubulin polymerization like colchicine and nocodazole’s destabilizing activity. Furthermore, we performed molecular docking studies in order to visualize the interactions between MBIC and tubulin complex. Results showed that MBIC fits well into the colchicine binding site in β tubulin complex (Additional file 2: Figure S2). MBIC interacted specifically with valine 181 (hydrophobic amino acid) and lysine 352 (polar amino acid) via hydrogen bond with tubulin complex (Additional file 2: Figure S2).

Cyclin-dependent kinases (CDKs) are cell cycle regulators, which control cell cycle shifts [36] and any dysregulation of CDKs, results in unscheduled cell proliferation as well as genomic instability [37]. We showed that MBIC induced cell cycle arrest in G_2_-M phase (Fig. 2c), with up-regulation of CDK1 and Cyclin B1 protein levels (Fig. 4a). Activation of Cyclin B1-CDK1 complex is required for initiation of mitosis [38]. The Cyclin B1-CDK1 induce lamin phosphorylation and subsequently, disassemble nuclear envelope [39]. After all chromosome’s kinetochores are attached to microtubules, anaphase-promoting complex/cyclosome (APC/C) degrades/deactivates Cyclin B1 and CDK1, to reform the nuclear envelope and exit from mitosis [40]. Thus, the protein level of these two enzymes affects cell cycle progression [41]. Disruption of microtubule dynamics causes kinetochores attachment errors to mitotic spindles. Spindle assembly checkpoint (SAC) detects unattached kinetochores before entering into anaphase [42]. Also, chromosomal passenger complex (CPC) detects mis-attached kinetochores [43]. When SAC is activated, it inhibits anaphase-promoting complex/cyclosome (APC/C) and stops Cyclin B1-CDK1 degradation [40]. Western blot analysis indicated that one of SAC proteins, BubR1 is up-regulated after MBIC treatment (Fig. 4a). BubR1 prevents the transition from metaphase to anaphase and avoids mitotic exit until all kinetochores are bound to microtubules correctly [44]. Therefore, up-regulation of BubR1 indicates microtubule-kinetochore attachment defects that may be due to MBIC-induced disruption of microtubule dynamics. If these errors are not corrected during the mitotic arrest, SAC is likely to signal induction of programmed cell death by restricting mitotic kinases function and accessibility [10]. As a CPC component, Aurora B supports the stabilization of microtubules around chromosomes by detecting faulty attached microtubule-kinetochore and creating unattached kinetochore. Consequently, unattached kinetochore signals the activation of SAC to inhibit APC/C. Disruption of Aurora B impairs SAC’s activity [45]. We observed down-regulation of Aurora B protein level which indicates manipulation of SAC activity.

Microscopic examination of MBIC-treated cells shows that they enter mitosis but can’t form metaphase spindles because microtubules cannot polymerize. Apart from damaging the mitotic spindle, live cell imaging revealed that MBIC also arrests the cell cycle in mitosis and promotes apoptosis. There are two possible scenarios after MTAs- induced mitotic arrest. Cells can either die or exit mitosis without undergoing cytokinesis. If the mitotic checkpoint is overridden, these cancer cells will then enter an abnormal, tetraploid G_1_-like phase. In our present study, we observed that most HeLa cells died after the prolonged mitotic arrest, suggesting that mitotic cell death or mitotic catastrophe (MC), but not mitotic slippage occurs in MBIC-treated HeLa cells. In fact, these treated cells showed features of apoptotic-like cell death. MC is known as an abnormal form of mitosis. The last step of MC is characterized by the random formation of nuclear envelopes around random mis-segregated chromosomes (multi-nucleation) and incomplete chromosome condensation [46]. Based on the knowledge that abnormalities in mitosis finally cause cell death, therefore MC has been classified as a step leading to apoptosis; not a mode of cell death. This is based on the fact that MC has several hallmarks of apoptosis such as mitochondrial-dependent apoptosis [47]. It has been reported caspases activation is essential for MC termination [48, 49]. One of the possibilities that can trigger mitochondrial-dependant apoptosis is decreased protein level of Bcl-2 that may happen upon the occurrence of prolonged mitotic arrest [39, 50]. Therefore, we decided to clarify whether MBIC-induced apoptosis, includes mitochondrial-dependent apoptosis.

Previous studies have shown that nocodazole-induced Bcl-2 down-regulation represents a pre-apoptotic phase after prolonged deformation of microtubules [51]. Another study demonstrated that G-1103, a MTA, exhibited both cell cycle arrest and mitochondrial-dependant apoptosis in HeLa cells [52]. In living cells Bcl-2 proteins attaches to Bax protein and avoid it to form pro-apoptotic homodimers on mitochondria’s outer membrane [53]. Bax alters mitochondrial membrane potential (MMP, Δψm) and this alteration leads to the release of apoptotic factors into the cytosol, such as cytochrome c. Cytochrome c promotes the activation of caspases [19]. Our study revealed that MBIC treatment caused up-regulation of Bax and down-regulation of Bcl-2 proteins in HeLa cells (Fig. 4c). These results indicate that HeLa cells that are stuck in prolonged G_2_-M arrest, undergo mitochondria-dependent apoptosis. We observed a dose-dependent increase of cleaved caspase-3/7/9 and cleaved poly-ADP-ribose polymerase (PARP) in HeLa cells after MBIC treatment (Fig. 4e). Caspase-3/7 activation and PARP cleavages can cause DNA damage [54]. These breaks activate DNA damage response and that results in apoptosis [55].

We assumed that MBIC is unique for cancer therapy due to its synergistic effect in combination with few current chemotherapeutic drugs. Favorable outcomes of synergism are dose and toxicity reduction together with minimizing drug resistance [12, 28]. For instance, docetaxel has been successful in clinical trials when combined with drugs such as trastuzumab, cetuximab, capecitabine, augmerosen and FTIs, in treating lung, ovarian, breast, prostate and gastric cancers [56]. Another successful drug candidate in combination therapy is SNS-314. SNS-314 is a mitotic kinase inhibitor that displays a synergistic effect in combination with gemcitabine, docetaxel and vincristine [57]. We found that MBIC showed synergism with colchicine, nocodazole, paclitaxel and doxorubicin with combination index (CI) values of up to 97 % in cervical cancer cell proliferation inhibition (CI < 1). Among these drugs, the highest synergistic effect was observed in combination with colchicine (IC_50_ = 0.076 + 0.03 uM). These drug combinations might regulate different mechanisms or modes of action and therefore, raise the efficacy of treatments against cancer cell growth. Synergism between a stabilizer (paclitaxel) and a destabilizer (MBIC) may seem controversial in cancer therapy. However such actions have been reported previously. I.e. the roots and rhizomes of *Tacca* species contained microtubule stabilizer (Taccalonolides) and destabilizer (Taccabulin A) compounds. Despite the fact that these two compounds have opposite actions on microtubule polymerization, together they have been shown to exhibit a synergistic effect on the proliferation of HeLa cells. During mitosis in order to separate sister chromatids, mitotic spindles grow and shrink rapidly. Therefore, microtubule dynamics are 3.6-fold more rapid than during interphase. Therefore, stabilizer and destabilizer net overall effect interferes with microtubule dynamics in mitosis particularly against highly proliferative cancer cells [58].

## Conclusion

The results of the present study demonstrated that methyl 2-(5-fluoro-2-hydroxyphenyl)-1H- benzo[d]imidazole-5-carboxylate (MBIC) is cytotoxic against various human cancer cell lines. Our findings further showed that HeLa cancer cells undergo mitotic arrest and subsequent apoptosis following treatment with MBIC. Our collective data suggest that MBIC induced cell cycle arrest in G_2_-M phase, which causes a mitotic catastrophe by specifically targeting the microtubule system. In addition, MBIC demonstrated synergistic effects in combination with conventional drugs such as colchicine, nocodazole, paclitaxel and doxorubicin in cervical cancer treatment. These findings indicate the potential therapeutic value of MBIC in treating cervical cancer.

## Additional files

Additional file 1: Figure S1. MBIC displayed synergistic effect with selected conventional drugs. A. shows isobolograms that link two concentrations of combined drug in their equal activity points (inhibition). In this isobolograms, we can see equal effect (50 %, 75 %, and 90 % cytotoxicity) points of MBIC in combination with indicated conventional drugs. B. is fractional affected-combination index (Fa-CI) plots that show 0 to 100 % inhibitions of MBIC combined with indicated conventional drugs in quantitatively definition of effect (CI) value. The CI value is lower than 1 represents the synergistic effect of MBIC in combination with a conventional drug, wherein their overall effect is higher than the effect of each individual drug. CI vale equals 1, represents the additive or indifferent effect of MBIC in combination with conventional drugs. CI value above 1 represents the antagonistic effect of MBIC in combination with conventional drugs wherein their overall effect is less than the effect of each individual drug. (PDF 207 kb) Additional file 2: Figure S2. Molecular docking of MBIC with tubulin complex. Hydrogen bonds between amino acids of tubulin complex and MBIC. Molecular structure was visualized by UCSF Chimera 1.8.1 program. Figure (A) is showing ribbon representation of tubulin-MBIC complex. Figure (B) is showing hydrogen bonds (green line) of MBIC with surrounding amino acids residues which are included polar lysine 352 and hydrophobic valine 181 amino acids. (PDF 3179 kb)
