# Supplementary figures and images for: Targeting of tubulin polymerization and induction of mitotic blockage by Methyl 2-(5-fluoro-2-hydroxyphenyl)-1H-benzo[d]imidazole-5-carboxylate (MBIC) in human cervical cancer HeLa cell
Source: J Exp Clin Cancer Res. 2016 Mar 31;35:58. doi: 10.1186/s13046-016-0332-0 (PMC4815073; doi:10.1186/s13046-016-0332-0)

Supplementary figure 1.

A

B

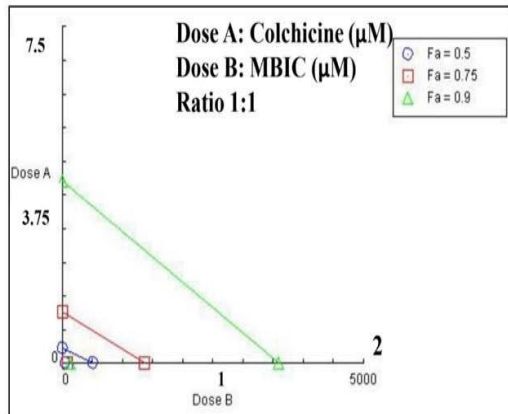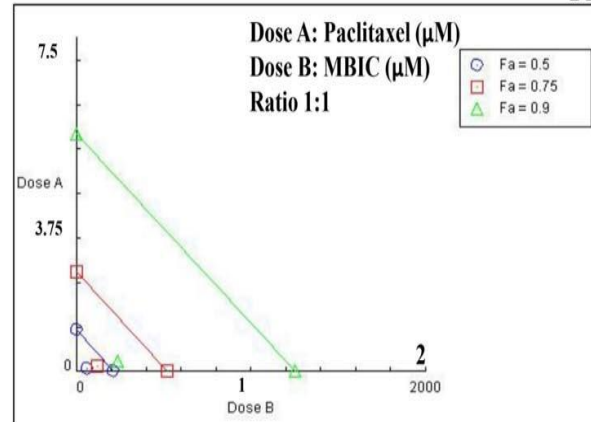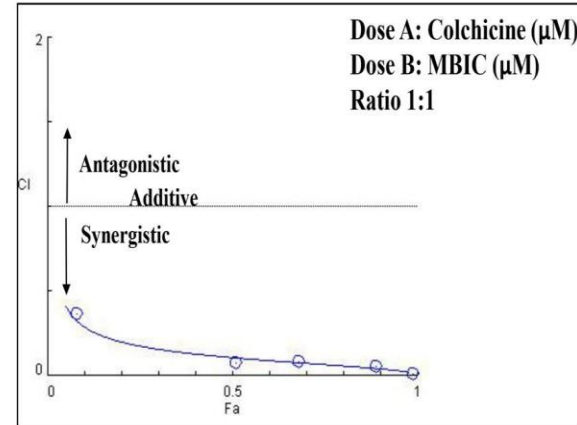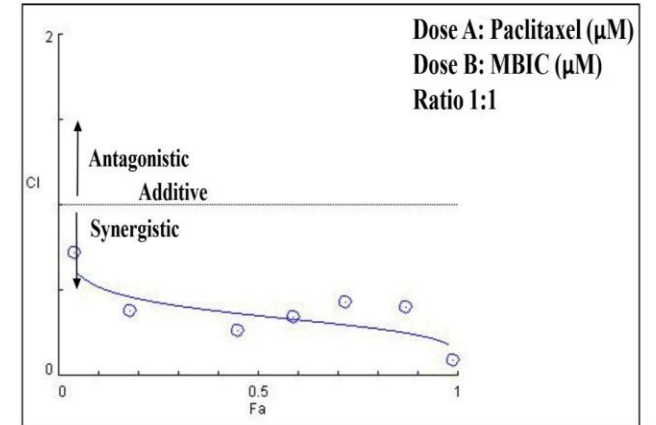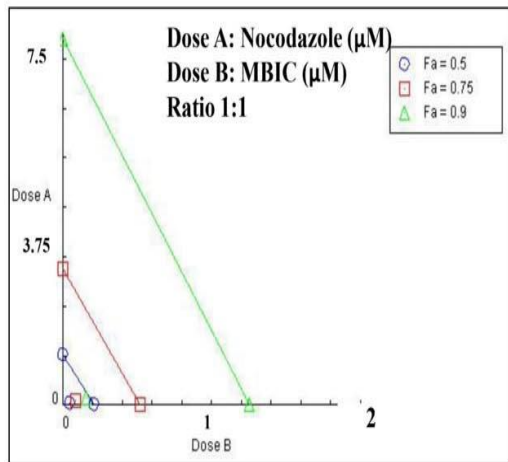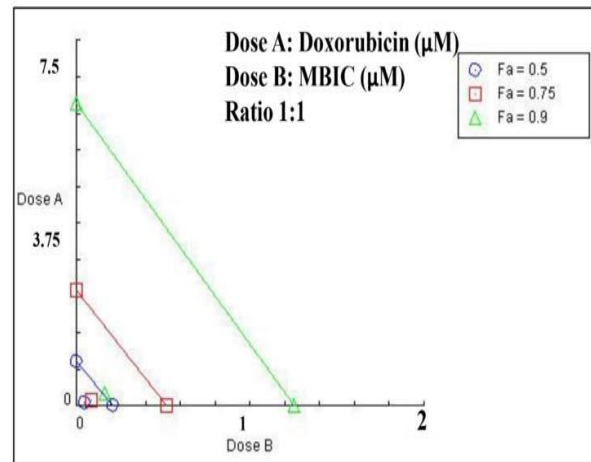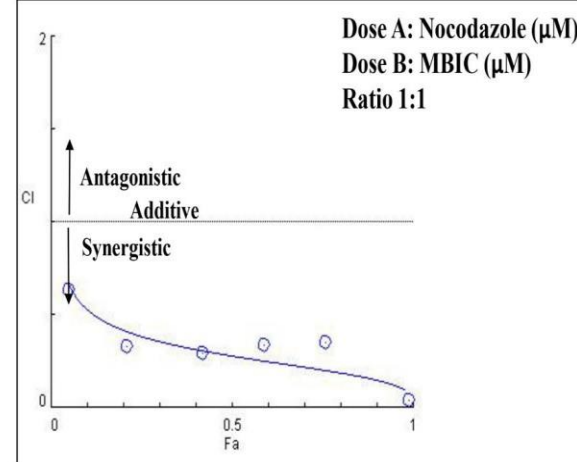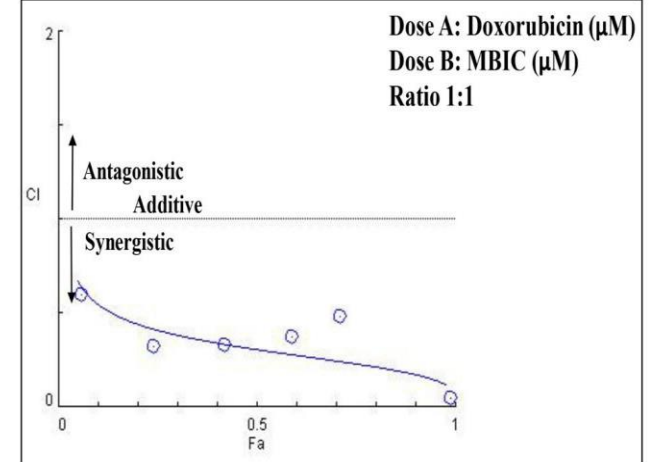

Supplement: Additional file 1: Figure S1. — MBIC displayed synergistic effect with selected conventional drugs. A. shows isobolograms that link two concentrations of combined drug in their equal activity points (inhibition). In this isobolograms, we can see equal effect (50 %, 75 %, and 90 % cytotoxicity) points of MBIC in combination with indicated conventional drugs. B. is fractional affected-combination index (Fa-CI) plots that show 0 to 100 % inhibitions of MBIC combined with indicated conventional drugs in quantitatively definition of effect (CI) value. The CI value is lower than 1 represents the synergistic effect of MBIC in combination with a conventional drug, wherein their overall effect is higher than the effect of each individual drug. CI vale equals 1, represents the additive or indifferent effect of MBIC in combination with conventional drugs. CI value above 1 represents the antagonistic effect of MBIC in combination with conventional drugs wherein their overall effect is less than the effect of each individual drug. (PDF 207 kb) [file 13046_2016_332_MOESM1_ESM.pdf]
