# Supplementary material for: Targeting of tubulin polymerization and induction of mitotic blockage by Methyl 2-(5-fluoro-2-hydroxyphenyl)-1H-benzo[d]imidazole-5-carboxylate (MBIC) in human cervical cancer HeLa cell
Source: J Exp Clin Cancer Res. 2016 Mar 31;35:58. doi: 10.1186/s13046-016-0332-0 (PMC4815073; doi:10.1186/s13046-016-0332-0)

Supplementary figure 2.

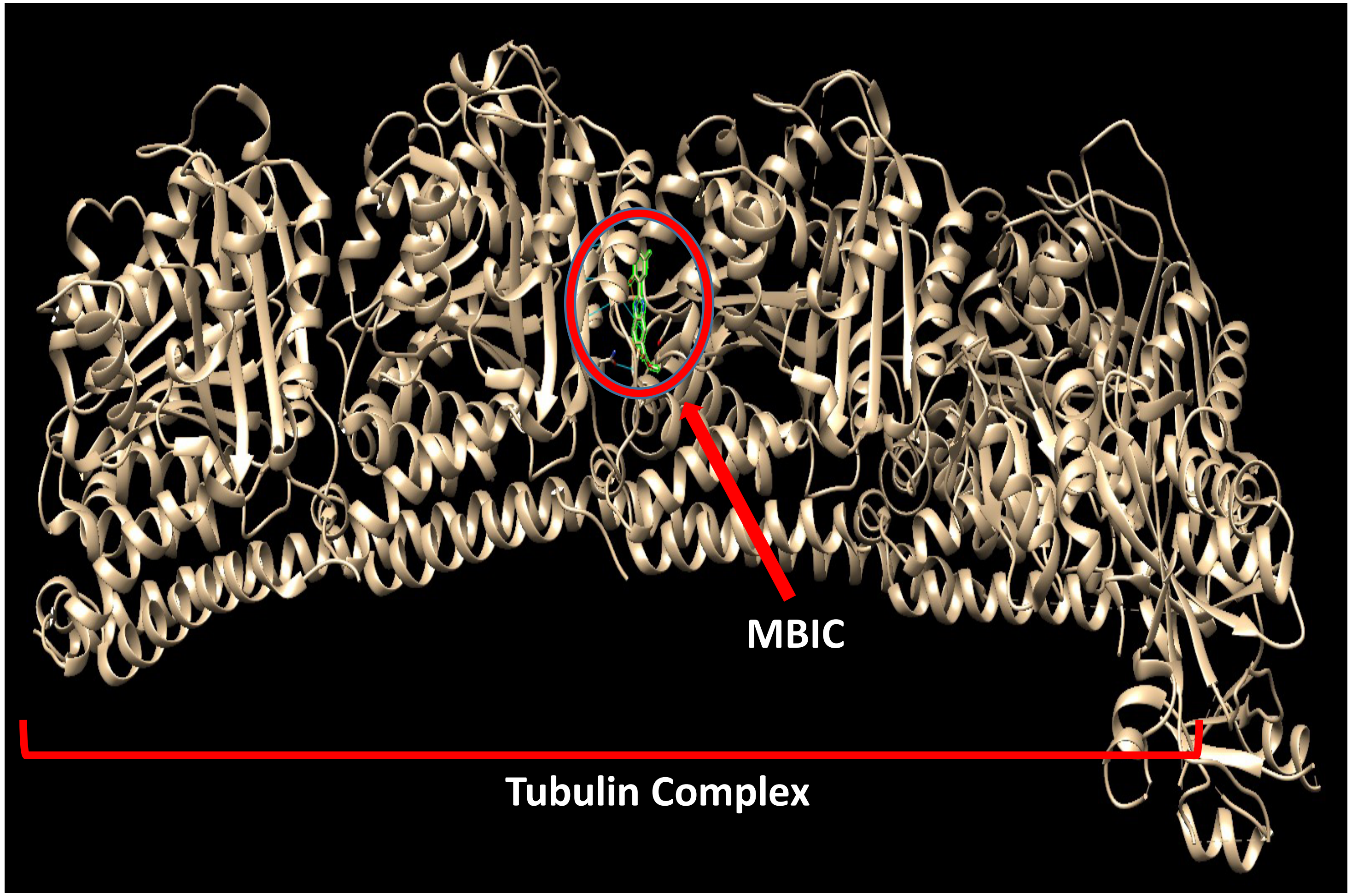

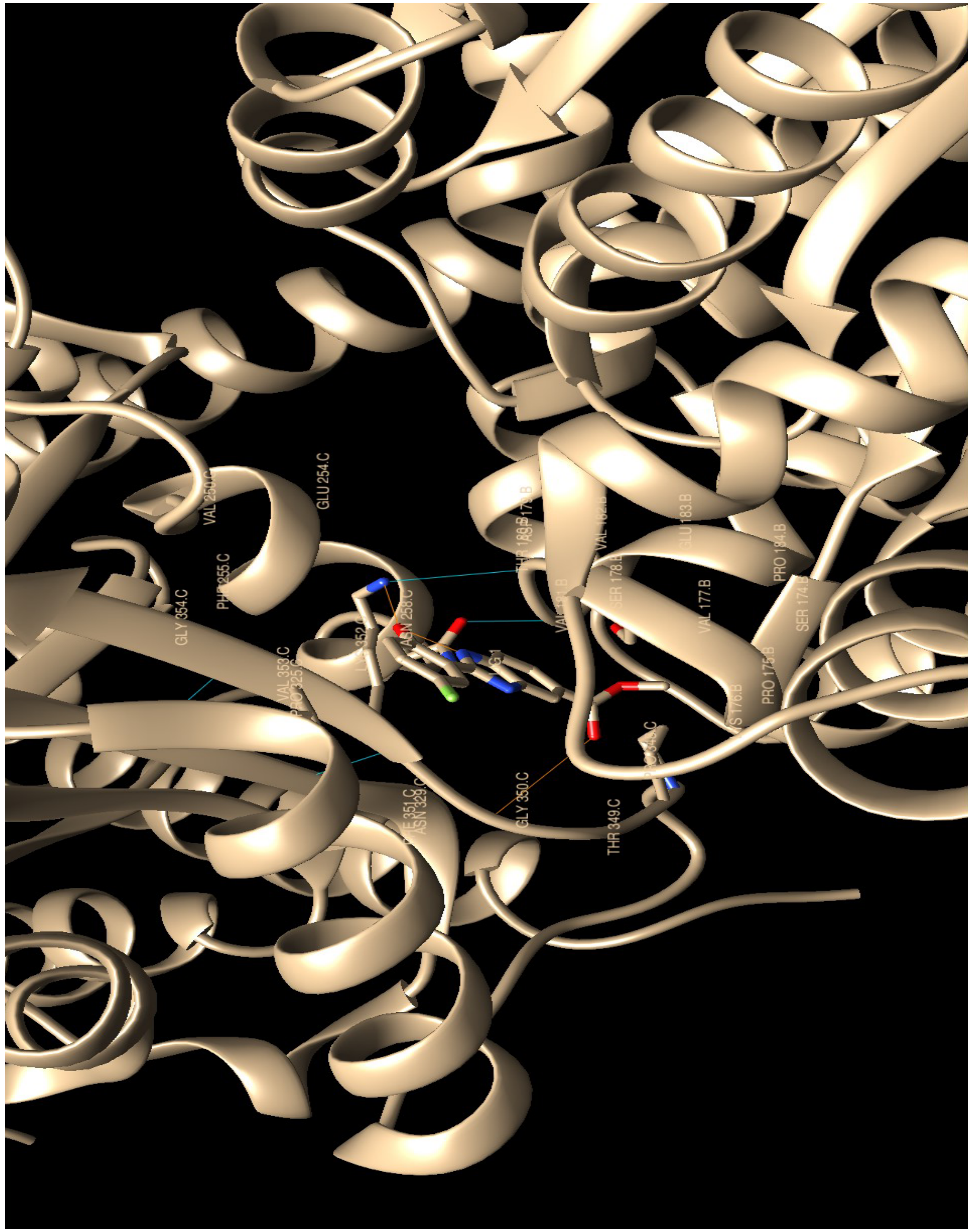

Supplement: Additional file 2: Figure S2. — Molecular docking of MBIC with tubulin complex. Hydrogen bonds between amino acids of tubulin complex and MBIC. Molecular structure was visualized by UCSF Chimera 1.8.1 program. Figure (A) is showing ribbon representation of tubulin-MBIC complex. Figure (B) is showing hydrogen bonds (green line) of MBIC with surrounding amino acids residues which are included polar lysine 352 and hydrophobic valine 181 amino acids. (PDF 3179 kb) [file 13046_2016_332_MOESM2_ESM.pdf]
